# Novel presentation of intraocular metastases in a patient with penile squamous cell carcinoma: a case report

**DOI:** 10.1186/s13256-020-02520-8

**Published:** 2020-10-23

**Authors:** Shanshan Li, Haifeng Zhao, Cui Qiu, Changfan Wu

**Affiliations:** Department of Ophthalmology, Yijishan Hospital of Wannan Medical College, Wuhu, 24000 China

**Keywords:** Penile cancer, Intraocular oncology, Choroidal metastases, Metastasis

## Abstract

**Background:**

The choroid is the most common site for intraocular tumor metastasis because of its abundant vascular supply. However, choroidal metastasis in penile cancer is highly unusual. Here, we report the first case of diagnosis of choroidal metastasis at presentation in a patient with penile squamous cell carcinoma.

**Case presentation:**

A 43-year-old Asian man with a 3-year history of penile cancer presented with metastasis in the right intraocular sites. Magnetic resonance imaging showed hyperintensity in the T1-weighted images and hypointensity in the T2-weighted images of the right eye. After enucleation of his right eye, histopathological analysis led to a diagnosis of metastatic, moderately differentiated penile squamous cell carcinoma.

**Conclusions:**

Penile cancer typically occurs as penile squamous cell carcinoma, and its most common metastatic sites are the inguinal lymph nodes. Hemorrhagic transfer of tumor cells is extremely rare, especially to intraocular sites. Intraocular metastatic tumors have a unique presentation on imaging, as observed on magnetic resonance imaging and histopathological analysis. This novel finding of intraocular metastasis in penile squamous cell carcinoma is of great significance to optic surgeons and oncologists as it has new implications in the diagnosis of and timely intervention for penile cancer metastasis.

## Background

Penile cancer (PC) is a rare cancer type in Europe, with an incidence of less than 1%. However, in parts of Asia, it may be account for as high as 10% of adult male cancers [1, 2]. PC initially spreads to the superficial and deep inguinal lymph nodes, and then follows a predictable pattern of distant metastasis via the lymphatic network [3–6]. However, tumor-cell dissemination through the blood is rare [3, 4, 7]. Research has shown that the lung and breast are the most frequent sites for PC metastasis, and other metastasis sites include the liver, brain, heart, skin, and bones [8–10]. Nevertheless, choroidal metastasis in PC is infrequent [3, 4]. Here, we present the first case report in the literature, to the best of our knowledge, of intraocular metastasis in a patient with PC, as confirmed with the patient’s clinical manifestation and magnetic resonance imaging (MRI) characteristics, as well as histopathology.

## Case presentation

On January 31, 2015, a 43-year-old Asian man with a 3-year history of progressively invasive PC presented with pain in his right eye. Our patient, who had been staged T4N3M1(TNM classification), had also lost his vision more than a month earlier. He was diagnosed as having metastasis in the bilateral inguinal lymph nodes and ipsilateral iliac nodes before systemic metastasis to his liver and lungs. His medical history was remarkable due to his several surgeries. He denied any family history of inherited diseases and psychological illness.

On presentation, his best corrected visual acuity was no light perception in his right eye and 20/20 in his left eye. His intraocular pressures were 13.0 mmHg and 11.0 mmHg in his right and left eye, respectively. For the right eye, the pupil dilated to 5 mm, and then the pupillary reaction disappeared. An external examination revealed mild proptosis and ocular movement in all directions. A dilated fundus examination of his right eye showed post equatorial retinal detachment with a black eminence and a pale optic disk. There were no obvious abnormalities in his left eye.

**Fig. 1:**
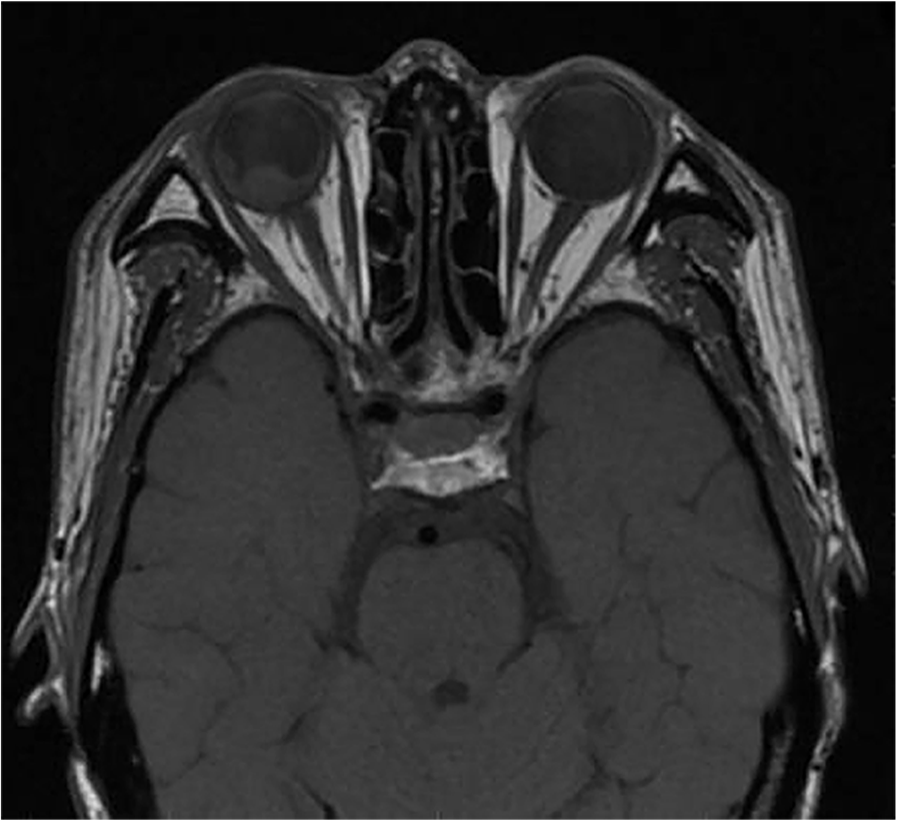
Transverse T1-weighted images revealed the presence of a right intraorbital fusiform mass with hyperintense on STIR sequence. The posterior wall was significantly thickened. The right optic nerve and peripheral fat gap were well-defined, and there was no obvious change in the extraocular muscle

**Fig. 2:**
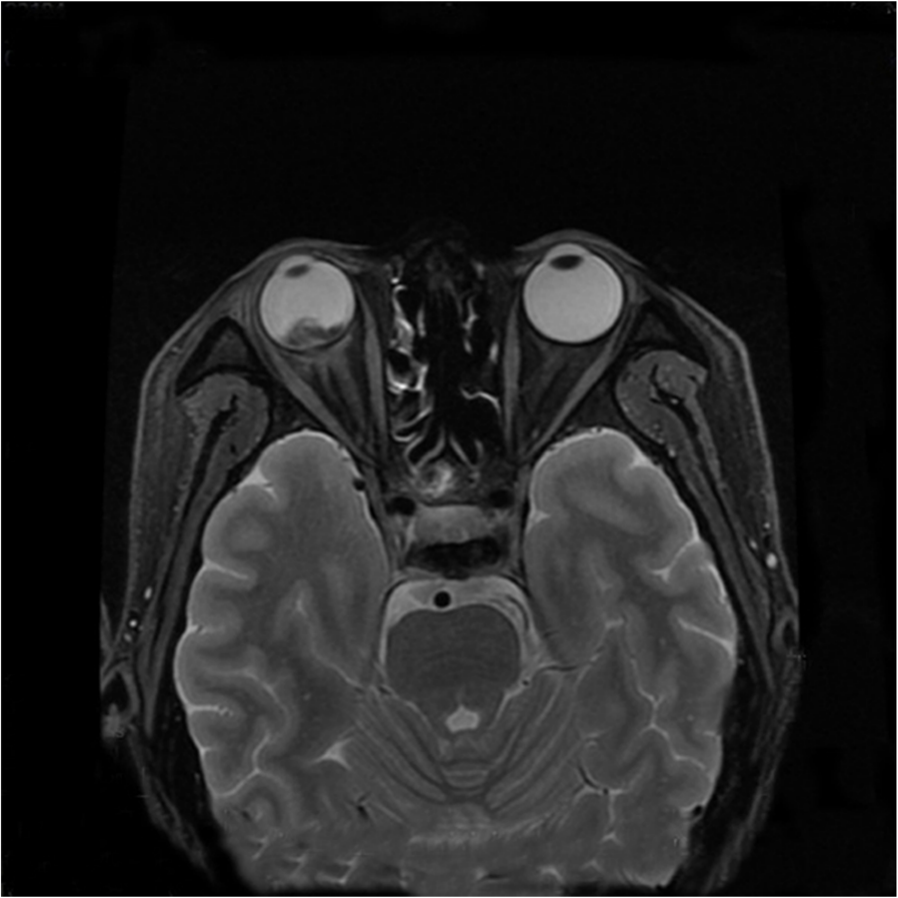
Orbital magnetic resonance imaging revealed fat suppression on T2-weighted images. The right ocular was proptosis. The strip-like hypointense in the posterior wall of the eyeball and flaky hyperintense in the intramuscular fat gap was observed

**Fig. 3:**
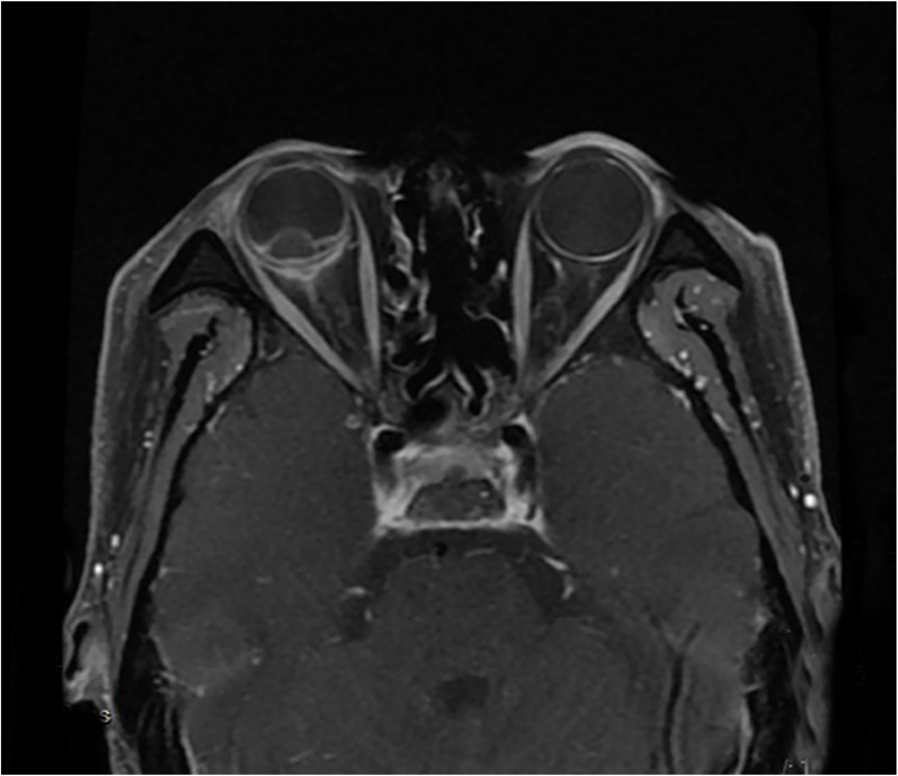
Transverse T1-weighted strengthened scanning images showed that the posterior segment of the right eye is characterized by “stratification” and irregular thickening of the posterior wall with reinforcement

An ophthalmic B-scan ultrasound showed retinal detachment with hemorrhage. Orbital MRI confirmed the thickening and strengthening of the right lateral wall, characteristics of metastatic carcinoma. The internal rectus and lateral rectus muscles were thickened and hardened, the 2-cm-long optic nerve was thickened, and its stump was invaded by the metastasis. The T1-weighted images of the MRI scans showed hyperintensity (Fig. 1), whereas the T2-weighted images showed hypointensity (Fig. 2). A contrast-enhanced MRI scan revealed inhomogeneous enhancement of the posterior wall (Fig. 3). The presence of lesions was associated with invasion of the optic nerve, choroid, and sclera by the metastatic cells.

**Fig. 4:**
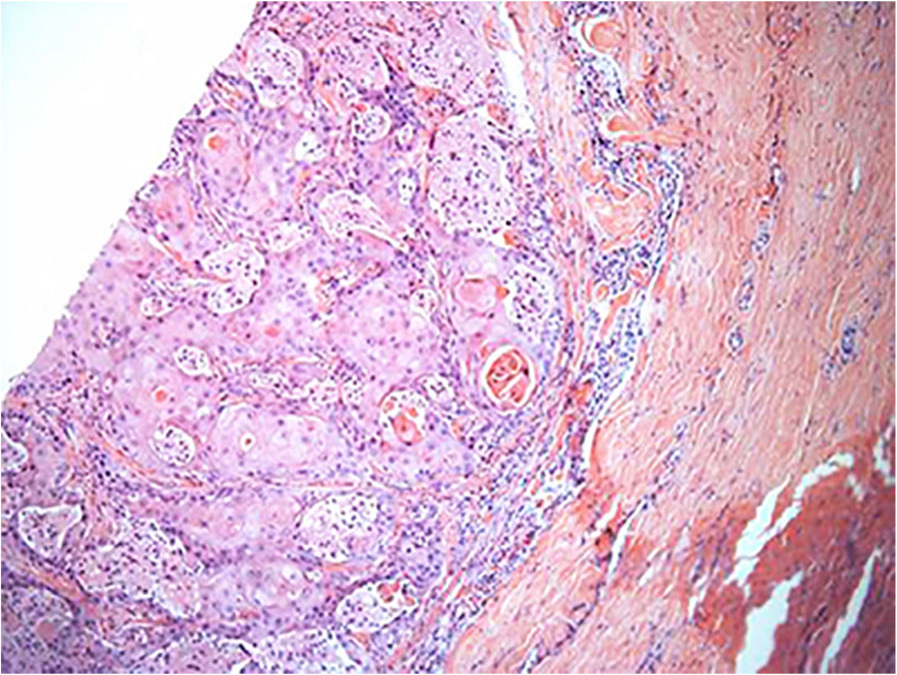
Histopathological examination after enucleation. Hematoxylin and eosin stain showed keratinized pearls and keratinized infiltrating growth in some areas of the tissues of well-differentiated squamous cell carcinoma. The cancer cells surrounded the optic nerve (×100 magnification)

**Fig. 5:**
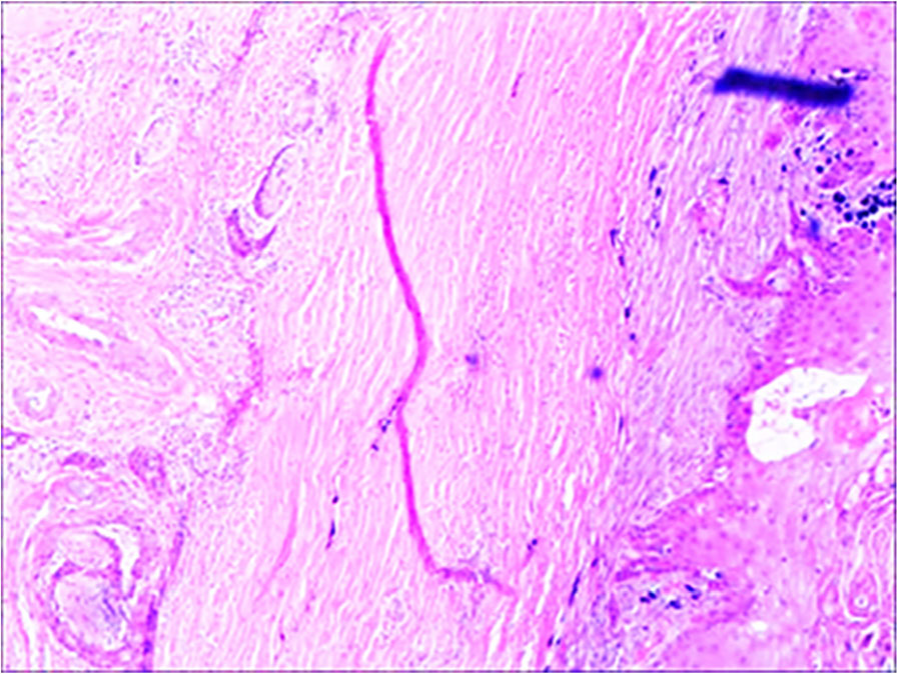
Hematoxylin and eosin stain indicated that the sclera, the choroid, and the optic nerve are infiltrated with cancer cells (×40 magnification)

The deep layer, including the choroid, was infiltrated by cancerous tissue. Considering his severe eye pain and irreversible loss of vision, our patient had undergone right eyeball enucleation under general anesthesia on February 3, 2015. This type of procedure is indicated for patients who have had severe eye trauma and for those patients experiencing severe eye pain with unrecoverable vision. His complete eyeball was observed intraoperatively. Histopathological examination led to a diagnosis of metastatic moderately differentiated penile squamous cell carcinoma that infiltrated the sclera, choroid, retina, optic nerve, and external intraocular sites. Hematoxylin-and-eosin staining of the entire eyeball cellular neoplasm showed keratin pearls and infiltrative growth of keratinized cells. Intercellular bridges were seen in the nests of moderately differentiated squamous carcinoma cells (Figs. 4 and 5).

Our patient received chemotherapy and radiotherapy during 6 months of follow-up, and then died due to brain metastasis.

## Discussion

Penile squamous cell carcinoma is a common urogenital cancer in Asia, which mainly spreads through the lymph nodes because of its rich lymphatic supply and relatively limited blood supply [7]. Metastasis to the lungs, bones, liver, and heart in PC have been described [3, 11, 12]. However, this is the first time that intraocular metastases have been diagnosed in a patient with PC.

Choroidal metastases are the most common intraocular tumors because of the choroid’s high vascular supply [2]. Choroidal metastases of primary tumors occur in 40–47% of all breast cancer patients and 21–29% of all lung cancer patients. Rare primary tumors in patients with PC also metastasize to the gastrointestinal tract, skin, and submandibular gland, and others [4]. In our patient, the extensive locoregional metastasis from the penis involved the inguinal and pelvic perivascular lymph nodes. Despite multiple partial penectomies and relative lymph node dissections, hematogenous metastasis occurred in the advanced stage, and intraocular structures were invaded.

Studies have shown that the left eye is more vulnerable to metastases than the right eye, as the left carotid artery starts directly from the aortic arch, whereas the right carotid artery arises from the innominate artery. However, metastasis in this patient occurred in his right eye before his left one, which was also shown by Kreusel *et al.* in 2008 [13]. Several studies have also shown that there were no differences in the sequence of metastasis of the eyes [5]. Additionally, ophthalmodynia may be caused by secondary glaucoma or tumor compression of the ciliary nerve. Ophthalmodynia in our patient was due to the latter. Furthermore, the black subretinal mass was associated with retinal hemorrhage caused by tumor invasion. A review by Arepalli *et al.* in 2015, found that choroidal metastases infrequently appear as brown-gray subretinal masses, usually associated with metastatic melanoma [4].

Choroidal metastasis may be commonly misdiagnosed as choroidal melanoma or sclerochoroidal calcification. Choroidal metastasis has a distinct pathology and manifests differently in imaging modalities than do other choroidal tumors. The MRI scan of our patient’s orbit showed a fusiform mass that presented as hyperintense on T1-weighted images and hypointense on T2-weighted images, in contrast to results from previous studies [4]. De Potter *et al.* in 1992 [14] described an uncommon presentation of choroidal metastasis on MRI that was similar to that in our patient, and it more accorded with a choroidal melanoma [4].

Diagnosis of ocular metastases is rare, as patients with eye metastases do not experience any symptoms or because general pain masks discomfort in the eyes. Unfortunately, no treatment currently exists for restoring patients’ vision lost by intraocular metastases. Therefore, physicians should pay attention to the possibility of intraocular metastases in PC and in other malignancies.

## Conclusions

In summary, this is the first case of PC metastasizing to intraocular sites, penetrating the choroid, and reaching the sclera. Orbital surgeons and oncologists should be cognizant of this novel site for PC metastasis.

## Data Availability

All of the data and materials will be available upon request to the corresponding author.

## Abbreviations

MRI: Magnetic resonance imaging
PC: Penile cancer
